# Swallowed a Gold Safety-Pin

**Published:** 1891-01

**Authors:** 


### SWALLOWED A GOLD SAFETY-PIN.

---

A patient of Dr. F. H. Wiggin, aged nine months, swallowed a gold pin, made something like a common safety-pin, an inch and a half long. The pin was open when swallowed, and was passed, pin point up, without harm, fifty-four hours later.—*Medical Record.*

### DR. A. W. CALHOUN'S TREATMENT FOR IRITIS.

*Local:*

Sulphate of zinc, alum and morphine . . . . aa gr. 2

*Constitutional:*

R. Iodide of potash . . . . . . oz. 1.
Bichloride of mercury . . . . . gr. 1
Peppermint water,
Pure water . . . . . . . aa oz. 3.

Sig.—Teaspoonful 3 times daily after meals.—*The Clinic.*

### ANÆMIA WITH AMENORRHŒA.

BY J. MILNER FOTHERGILL.

℞ Acidi arseniosi, gr. j.

Ferri sulphat. exciccat., ʒ ss.

Pulv. pip. nigr. ʒ j.

Pil. alœs et myrrhae, ʒ j.

M.—Et. div. in pil. No. xl.

Sig. One twice a day after meals.

